# Adolescent fluoxetine treatment mediates a persistent anxiety-like outcome in female C57BL/6 mice that is ameliorated by fluoxetine re-exposure in adulthood

**DOI:** 10.1038/s41598-021-87378-6

**Published:** 2021-04-08

**Authors:** Francisco J. Flores-Ramirez, Anapaula Themann, Jorge A. Sierra-Fonseca, Israel Garcia-Carachure, Samuel A. Castillo, Minerva Rodriguez, Omar Lira, Joshua Preciado-Piña, Brandon L. Warren, Alfred J. Robison, Sergio D. Iñiguez

**Affiliations:** 1 Department of Psychology, The University of Texas at El Paso, 500 West University Avenue, El Paso, TX 79968 USA; 2 Department of Pharmacodynamics, University of Florida, Gainesville, FL USA; 3 Department of Physiology, Michigan State University, East Lansing, MI USA

**Keywords:** Anxiety, Neurotrophic factors, Prefrontal cortex, Drug safety

## Abstract

The objective of this study was to evaluate whether juvenile fluoxetine (FLX) exposure induces long-term changes in baseline responses to anxiety-inducing environments, and if so, whether its re-exposure in adulthood would ameliorate this anxiety-like phenotype. An additional goal was to assess the impact of adolescent FLX pretreatment, and its re-exposure in adulthood, on serotonin transporters (5-HTT) and brain-derived-neurotrophic-factor (BDNF)-related signaling markers (TrkB-ERK1/2-CREB-proBDNF-mBDNF) within the hippocampus and prefrontal cortex. To do this, female C57BL/6 mice were exposed to FLX in drinking water during postnatal-days (PD) 35–49. After a 21-day washout-period (PD70), mice were either euthanized (tissue collection) or evaluated on anxiety-related tests (open field, light/dark box, elevated plus-maze). Juvenile FLX history resulted in a persistent avoidance-like profile, along with decreases in BDNF-signaling markers, but not 5-HTTs or TrkB receptors, within both brain regions. Interestingly, FLX re-exposure in adulthood reversed the enduring FLX-induced anxiety-related responses across all behavioral tasks, while restoring ERK2-CREB-proBDNF markers to control levels and increasing mBDNF within the prefrontal cortex, but not the hippocampus. Collectively, these results indicate that adolescent FLX history mediates neurobehavioral adaptations that endure into adulthood, which are indicative of a generalized anxiety-like phenotype, and that this persistent effect is ameliorated by later-life FLX re-exposure, in a prefrontal cortex-specific manner.

## Introduction

Anxiety disorders are among the most commonly diagnosed psychiatric illnesses in children and adolescents^1,2^. Young people with anxiety disorders display increased risk adverse outcomes, including academic failures, family and social dysfunction, and comorbid mood-related illnesses such as major depression^3,4^. Interestingly, while selective serotonin reuptake inhibitor (SSRI) medications were first introduced for the treatment of major depression, they also represent the preferred pharmacotherapeutic approach for anxiety disorders^5,6^. Indeed, SSRIs like fluoxetine (FLX) are effective for the management of pediatric anxiety, with an overall response rate almost twice as great when compared to placebo^7^. FLX’s best known mechanism of action is via the blockade of serotonin reuptake transporters (5-HTT), indirectly increasing serotonin levels at the synapse—a mechanism that may underlie its therapeutic outcomes^8^.

Given the implication of serotonin in neurodevelopmental processes, such as neuronal proliferation, differentiation, migration, and synaptogenesis^9,10^, as well as the link between 5-HTTs and mood-related illnesses^8^, it is possible that ontogenic exposure to SSRIs may lead to unexpected effects in later life^11^. Indeed, at the preclinical level, accumulating studies indicate that juvenile FLX exposure results in long-term changes in memory performance^12,13^, altered responsivity to despair measures and drugs of abuse^14,15^, along with increased reactivity to anxiety-eliciting situations^16–19^. These enduring effects, collectively, highlight the need for caution when exposing young individuals to SSRIs.

The neurobiological mechanisms underlying early-life SSRI-induced behavioral changes in adulthood are not well understood^17^. However, some studies point to long-term FLX-induced alterations in 5-HTTs and brain derived neurotropic factor (BDNF)-related signaling molecules across different brain regions^15,20,21^, including the prefrontal cortex and hippocampus^22,23^; structures that modulate affect-related behavior^24–27^. Indeed, within these brain regions, SSRIs reverse the stress-induced decreases of BDNF and its receptor TrkB (BDNF-Tropomyosin receptor kinase B)^28^, as well as the expression of ERK1/2 (extracellular signal-regulated kinase 1/2)^29^, and the transcription factor CREB (cAMP response element-binding protein)^30^; ultimately, facilitating neuronal growth and survival^31^.

**Figure 1:**
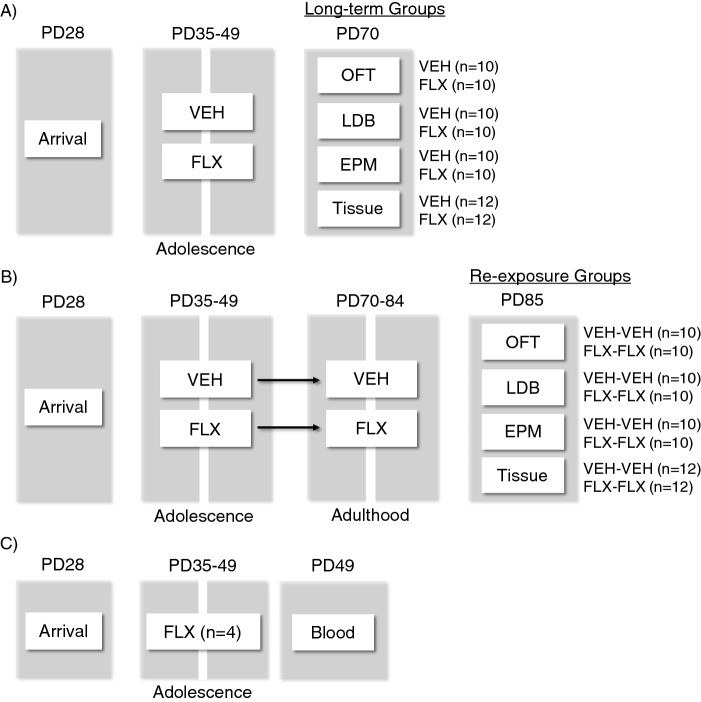
Timeline of juvenile fluoxetine (FLX) treatment and experimental procedures. (**A**) Postnatal day (PD)-28 female C57BL/6 mice arrived to our animal colony. One week later (PD35), they were randomly assigned to receive FLX in their drinking water (250 mg/l) for 15 consecutive days (PD35-49), or water alone (vehicle, VEH). After a 21-day washout period (PD70), separate groups of mice were tested on the open field test (OFT), light/dark box (LDB), elevated plus maze (EPM), or euthanized for tissue extraction (Long-term groups). (**B**) Separate groups of PD28 mice arrived to our animal colony. One-week later (PD35) they were exposed to VEH or FLX in their drinking water (PD35-49). After a 21-day washout period (PD70), FLX or VEH was re-administered for two additional weeks (PD70-84). Twenty-four h after FLX or VEH re-exposure (PD85), mice were either tested on the OFT, LDB, EPM, or euthanized for tissue extraction (Re-exposure groups). (**C**) Blood was collected from a separate group of animals on PD49 to evaluate serum FLX concentrations after exposure to FLX in their drinking water for 15 days.

It is noteworthy that studies assessing FLX’s mechanism of action and efficacy, as well as its short- and long-term neurobehavioral effects, have primarily utilized male subjects. The exclusion of females in such experimental approaches is limiting and problematic, given the well-established difference between the sexes in drug efficacy, sensitivity, and safety^32,33^. Moreover, clinical data demonstrate that women are more likely than men to be diagnosed with an anxiety-related disorder, a sex-difference that becomes apparent by mid-adolescence^34,35^. Consequently, young girls are more likely to be prescribed with psychotropic medications like FLX^36^. To address this gap in the literature, the goal of this study is to examine whether juvenile FLX exposure increases reactivity in paradigms commonly used to evaluate anxiety-related behavior^37^ in adult female C57BL/6 mice, and if so, whether FLX re-exposure normalizes the SSRI-induced avoidance-like behavioral response. Furthermore, to evaluate the enduring effects of adolescent FLX-pretreatment, and its re-exposure in adulthood, on 5-HTTs and BDNF-related molecules (TrkB, ERK1/2, CREB) within the prefrontal cortex and hippocampus (Fig. 1).

## Results

### Long-term effects of adolescent FLX treatment and re-exposure in adulthood on the OFT

**Figure 2:**
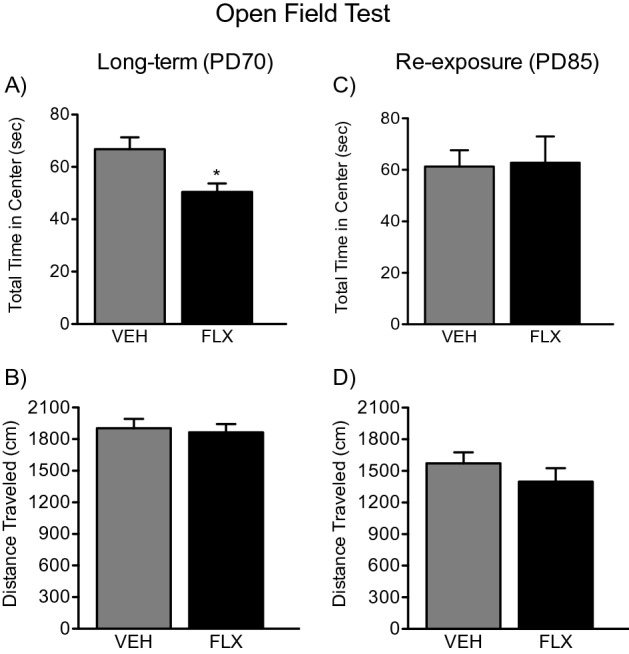
Long-term effects of juvenile fluoxetine (FLX) treatment, and its re-exposure in adulthood, on the open field test. (**A**) FLX pretreatment during adolescence (PD35-49) resulted in a decrease in the time spent in the center area of the open field arena at PD70 (Long-Term). (**B**) FLX pretreatment did not influence total distance traveled during the 5-min session of the open field test. (**C**) Re-exposure to FLX in adulthood (PD70-84) ameliorated the long-lasting anxiety-like effect of adolescent FLX pre-exposure, as no differences in total time spent in the center between the groups were observed. (**D**) At PD85, no differences in distance traveled were apparent between the experimental groups. Data are presented as mean ± SEM. *p < 0.05.

Figure 2A,B displays the effects of juvenile FLX exposure (PD35-49) on the OFT in adulthood. PD70 female mice pretreated with FLX (n = 10) displayed decreased time in the center zone of the arena when compared to VEH-pretreated (n = 10) controls (t_18_ = 2.91, *p* < 0.05; Fig. 2A). No differences in locomotor activity (total distance traveled) were observed between the groups (*p* > 0.05; Fig. 2B).

Figure 2C,D displays the effects of FLX re-exposure (PD70-84) on the OFT in adult female mice (PD85) with adolescent FLX history (PD35-49). When compared to VEH-pretreated controls (n = 10), PD85 female mice with juvenile antidepressant history and re-exposure in adulthood (n = 10) did not display differences in time spent in the center zone of the OFT (*p* > 0.05; Fig. 2C), or differences in total distance traveled during the 5 min test (*p* > 0.05; Fig. 2D).

### Long-term effects of adolescent FLX treatment and its re-exposure in adulthood on the LDB test

**Figure 3:**
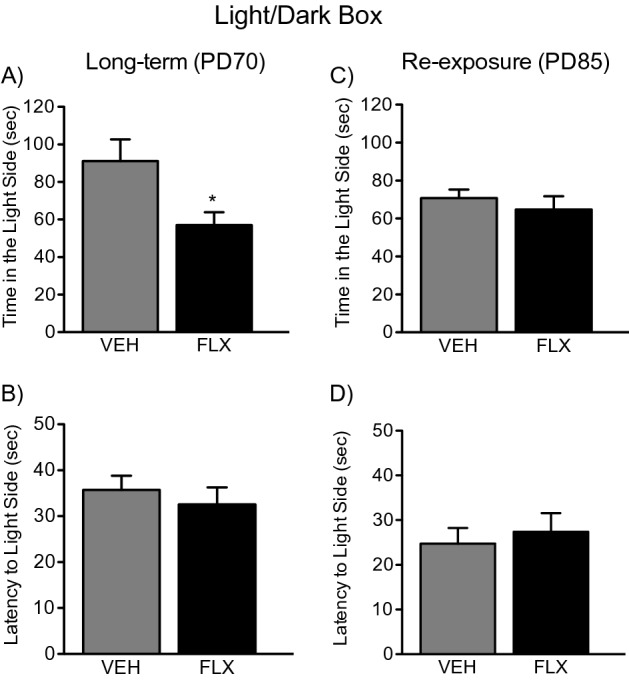
Long-term effects of juvenile fluoxetine (FLX) treatment, and its re-exposure in adulthood, on the light/dark box. (**A**) Juvenile FLX pretreatment (PD35-49) led to a significantly decreased time spent in the lighted-side of the light/dark box at PD70 (Long-Term), when compared to water (VEH)-pretreated controls. (**B**) When compared to VEH-pretreated controls, juvenile FLX pretreatment did not influence the time it took the animals to first enter the lighted-side of the apparatus. (**C**) FLX re-exposure in adulthood (PD70-84) ameliorated the long-lasting anxiety-like effect of juvenile FLX pretreatment, as no differences in total time spent in the center between the groups were observed. (**D**) At PD85, no differences in the time it took for animals to first enter the lighted-side of the apparatus were apparent between the experimental groups. Data are presented as mean ± SEM. *p < 0.05.

Figure 3A,B shows the effects of adolescent FLX exposure (PD35-49) on the LDB in adulthood (PD70). When compared to VEH-pretreated controls (n = 10), FLX-pretreated mice (n = 10) spent significantly less time in the light side of the apparatus (t_18_ = 2.55, *p* < 0.05; Fig. 3A). No differences in the latency to enter the lighted side of the box were observed between the groups (*p* > 0.05; Fig. 3B).

Figure 3C,D displays the effects of FLX re-exposure (PD70-84) on the LDB in adult female mice (PD85) with adolescent FLX history (PD35-49). When compared to VEH-pretreated controls (n = 10), PD85 mice with juvenile FLX history and re-exposure in adulthood (n = 10) did not differ in time spent in the lighted side of the apparatus (*p* > 0.05; Fig. 3C). No differences in latency to enter the lighted side of the box were observed between the groups (*p* > 0.05; Fig. 3D).

### Long-term effects of adolescent FLX treatment and its re-exposure in adulthood on the EPM

**Figure 4:**
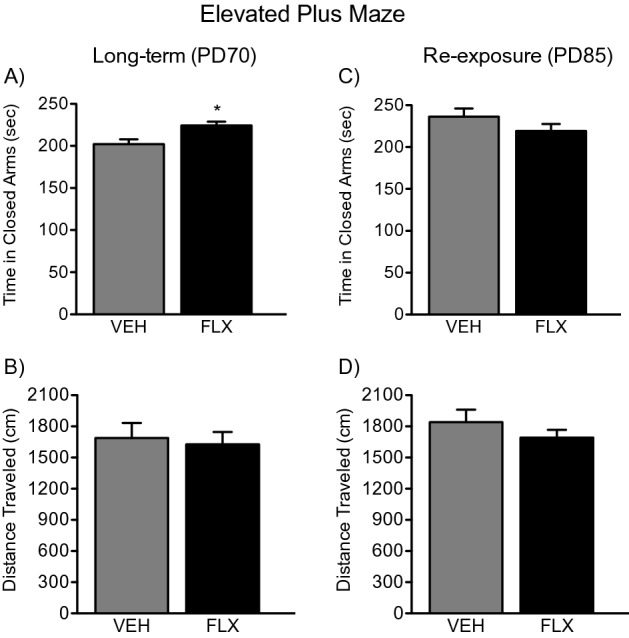
Long-term effects of juvenile fluoxetine (FLX) treatment, and its re-exposure in adulthood, on the elevated plus maze. (**A**) Adolescent FLX pretreatment (PD35-49) resulted in a significant increase in time spent in the closed arms of the maze at PD70 (Long-Term), when compared to water (VEH)-treated controls. (**B**) No differences in distance traveled during the 5-min test were observed between the groups. (**C**) Re-exposure of FLX in adulthood (PD70-84) ameliorated the long-lasting anxiety-like effect of juvenile FLX pre-exposure since no differences in total time spent in the closed arms were noted between the groups (PD85). (**D**) No differences in distance traveled were observed between the experimental groups. Data are presented as mean ± SEM. *p < 0.05.

Figure 4A,B shows the effects of adolescent FLX history (PD35-49) on anxiety-like behavior in the EPM in adulthood (PD70). A Student’s t test indicated that FLX-pretreated mice (n = 10) spent significantly more time in the closed arms of the maze when compared to VEH-pretreated (n = 10) controls (t_18_ = 3.04, *p* < 0.05; Fig. 4A). No differences in locomotor activity (distance traveled) were observed between the groups (*p* > 0.05; Fig. 4B).

Figure 4C,D displays the effects of FLX re-exposure (PD70-84) on the EPM in adult female mice (PD85) with adolescent FLX history (PD35-49). When compared to VEH-pretreated controls (n = 10), PD85 mice with juvenile FLX history and re-exposure in adulthood (n = 10) did not differ in time spent in the closed arms of the EPM (*p* > 0.05; Fig. 4C). Lastly, no differences in distance traveled were observed between the groups (*p* > 0.05; Fig. 4D).

### Long-term effects of adolescent FLX treatment and re-exposure in adulthood on the hippocampal expression of 5-HTTs and BDNF-related signaling molecules

**Figure 5:**
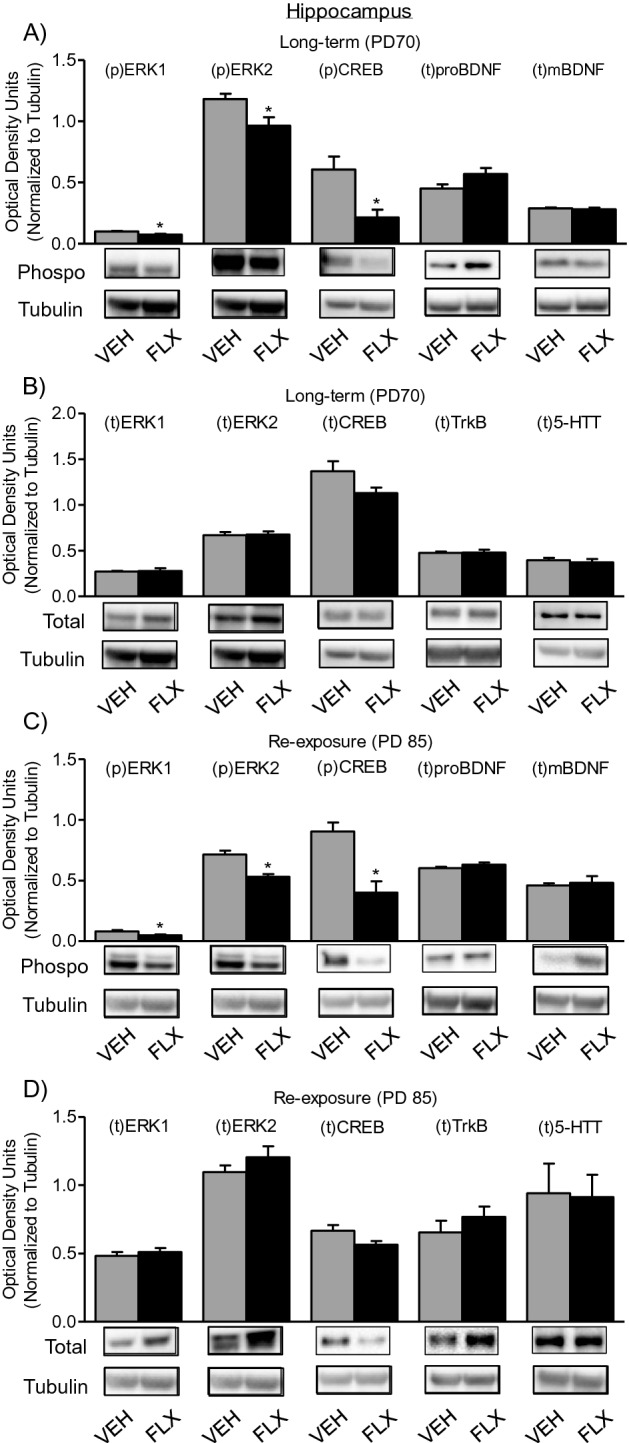
Long-term effects of juvenile FLX treatment, and its re-exposure in adulthood, on ERK1/2, CREB, BDNF TrkB, and 5-HTT proteins in the hippocampus of female C57BL/6 mice. (**A**) Adolescent FLX pretreatment (PD35-49) significantly decreased phosphorylated (p) ERK1, (p)ERK2, and (p)CREB without altering total (t) proBDNF or mBDNF at PD70 (Long-term), when compared to water (VEH)-treated controls. (**B**) No differences in (t)ERK1/2, (t)CREB, (t)TrkB, or (t)5-HTT were noted between the groups 21-days after adolescent FLX pretreatment. (**C**) Re-exposure to FLX in adulthood (PD70-84), in female mice with adolescent FLX history, decreased phosphorylated (p) levels of ERK1, ERK2, and CREB, with no differences in total proBDNF or mBDNF, 24 h after the end of treatment (PD85). (**D**) No differences in total ERK1/2, CREB, TrkB, or 5-HTT were observed on PD85, as a function of juvenile FLX exposure (PD35-49) and re-exposure in adulthood (PD70-84). Western blots of hippocampal tissue, with α-Tubulin as loading control, were performed under identical protocols. Representative images were cropped from different blots (Supplemental Figure S1). Data are presented as mean ± SEM. *p < 0.05.

Figure 5A shows the enduring effects of adolescent FLX history (PD35-49) on the phosphorylation of signaling markers ERK1/2 and CREB, as well as total levels of proBDNF and mBDNF within the hippocampus of PD70 female C57BL/6 mice (Long-term group). When compared to VEH-pretreated (n = 12) controls, FLX-pretreated mice (n = 12) displayed significant decreases in pERK1 (t_22_ = 3.04, *p* < 0.05), pERK2 (t_22_ = 2.64, *p* < 0.05), as well as pCREB (t_22_ = 3.18, *p* < 0.05), with no differences in total protein levels of proBDNF (*p* > 0.05) or mBDNF (*p* > 0.05).

Figure 5B shows the enduring effects of adolescent FLX exposure (PD35-49) on total levels of ERK1/2, CREB, TrkB, and 5-HTT within the hippocampus of PD70 female C57BL/6 mice (Long-term group). No differences in the total levels of ERK1/2, CREB, TrkB, or 5-HTT (*p* > 0.05) were observed in the hippocampus of adult female mice as a function of FLX exposure during adolescence.

Figure 5C shows the effects of adolescent FLX exposure (PD35-49) and re-exposure in adulthood (PD70-84) on phosphorylation of ERK1/2, CREB, and total levels of proBDNF and mBDNF within the hippocampus of PD85 female C57BL/6 mice (Re-exposure group). Here, animals with FLX history displayed persistent decreases in phosphorylated levels of ERK1 (t_22_ = 3.01, *p* < 0.05), ERK2 (t_22_ = 4.80, *p* < 0.05), as well as CREB (t_22_ = 4.24, *p* < 0.05), with no changes in total levels of proBDNF or mBDNF (*p* > 0.05).

Figure 5D shows the effects of adolescent FLX exposure (PD35-49) and re-exposure (PD70-84) on total protein levels of ERK1/2, CREB, TrkB, and 5-HTT within the hippocampus of PD85 female C57BL/6 mice (Re-exposure group). No differences in the total levels of ERK1, ERK2, CREB, TrkB, or 5-HTT (*p* > 0.05) were apparent in the hippocampus of adult female mice, as a function of juvenile FLX exposure and adult re-exposure.

### Long-term effects of adolescent FLX treatment and re-exposure in adulthood on the expression of 5-HTTs and BDNF-related signaling molecules in the prefrontal cortex

**Figure 6:**
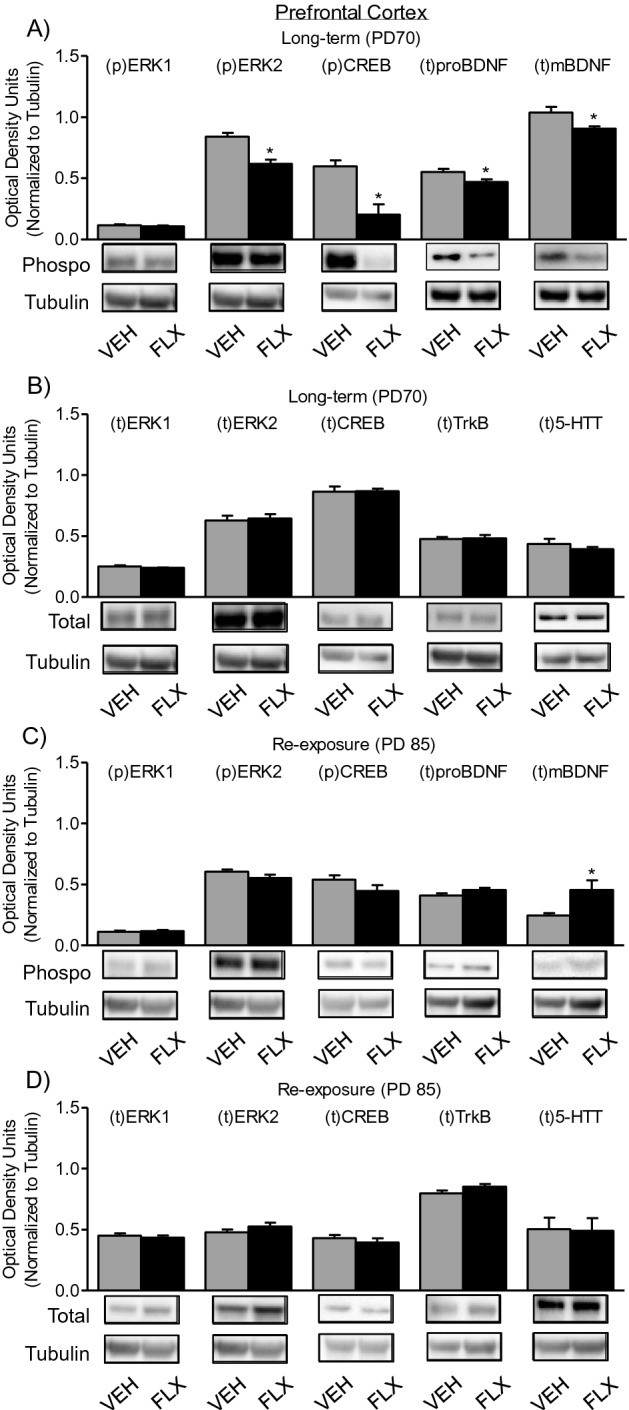
Long-term effects of juvenile FLX treatment, and its re-exposure in adulthood, on ERK1/2, CREB, BDNF TrkB, and 5-HTT proteins in the prefrontal cortex of female C57BL/6 mice. (**A**) Adolescent FLX pretreatment (PD35-49) resulted in significant decreases in phosphorylated (p) ERK2, (p)CREB, (t)proBDNF, and (t)mBDNF at PD70 (Long-term), when compared to water (VEH)-treated controls (**B**) No significant differences in total protein levels of ERK1/2, CREB, TrkB were apparent 21-days after juvenile FLX treatment. (**C**) Re-exposure to FLX in adulthood (PD70-84) normalized (p)ERK, (p)CREB, and (t)proBDNF, and significantly increased (t)mBDNF, 24 h after the end of antidepressant re-exposure (PD85). (**D**) No differences in (t)ERK1/2, (t)CREB, (t)TrkB, or (t)5-HTT were observed on PD85, as a function of juvenile FLX exposure (PD35-49) and re-exposure in adulthood (PD70-84). Western blots of prefrontal cortex tissue, with α-Tubulin as loading control, were performed under identical protocols. Representative images were cropped from different blots (Supplemental Figure S2). Data are presented as mean ± SEM. *p < 0.05.

Figure 6A shows the enduring effects of adolescent FLX history (PD35-49) on phosphorylation of signaling markers ERK1/2 and CREB, as well as total levels of proBDNF and mBDNF within the prefrontal cortex of PD70 female C57BL/6 mice (Long-term group). When compared to VEH-pretreated (n = 12) controls, FLX-pretreated mice (n = 12) displayed significant decreases in pERK2 (t_22_ = 5.07, *p* < 0.05), pCREB (t_22_ = 3.99, *p* < 0.05), and total protein levels of proBDNF (t_22_ = 2.44, *p* < 0.05) and mBDNF (t_22_ = 2.50, *p* < 0.05). No differences in phosphorylation of ERK1 were noted between the groups (*p* > 0.05).

Figure 6B shows the enduring effects of adolescent FLX exposure (PD35-49) on total protein levels of ERK1/2, CREB, TrkB, and 5-HTT within the prefrontal cortex of PD70 female C57BL/6 mice (Long-term group). No differences in the total levels of ERK1/2, CREB, TrkB, or 5-HTT (*p* > 0.05) were observed in the adult prefrontal cortex of female mice, as a function of FLX history during adolescence.

Figure 6C shows the effects of adolescent FLX exposure (PD35-49) and re-exposure in adulthood (PD70-84) on phosphorylation of ERK1/2 and CREB, and total protein levels of proBDNF and mBDNF within the prefrontal cortex of PD85 female C57BL/6 mice (Re-exposure group). Here, no differences in phosphorylation of ERK1/2, CREB, or total levels of proBDNF (*p* > 0.05, respectively) were observed. However, a significant increase in total protein of mBDNF (t_22_ = 2.57, *p* < 0.05) was apparent in the prefrontal cortex of PD85 female mice with juvenile FLX history and re-exposure in adulthood, when compared to VEH controls.

Figure 6D shows the effects of adolescent FLX exposure (PD35-49) and re-exposure in adulthood (PD70-84) on total protein levels of ERK1/2, CREB, TrkB, and 5-HTT within the prefrontal cortex of PD85 female C57BL/6 mice (Re-exposure group). Here, no differences in total protein levels of ERK1/2, CREB, TrkB, or 5-HTT (*p* > 0.05) were apparent in the prefrontal cortex of PD85 female mice, as a function of juvenile FLX exposure and its re-exposure in adulthood.

### Serum FLX levels in adolescent mice

**Figure 7:**
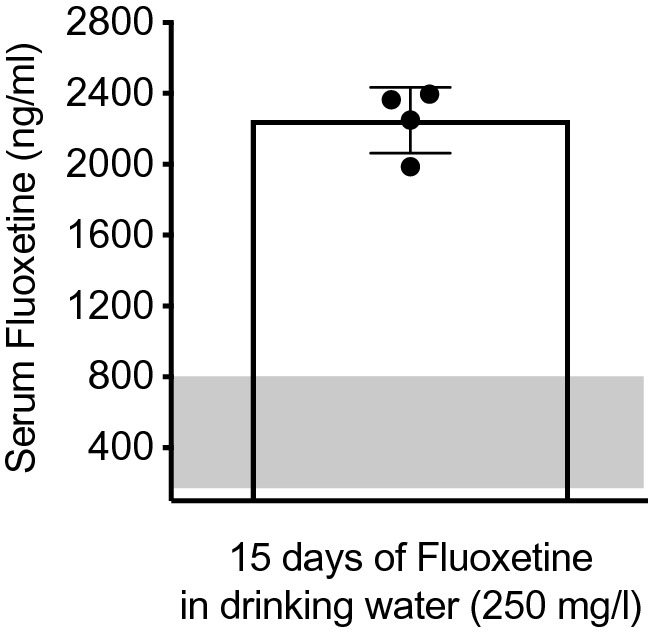
Plasma fluoxetine levels. Plasma fluoxetine levels are shown in ng/ml for adolescent (postnatal day 49) female C57BL/6 mice receiving 250 mg/l of fluoxetine in their drinking water (for 15 days). The gray area displays plasma fluoxetine levels in humans taking 20–80 mg fluoxetine per day^63,65^.

FLX blood serum was assessed in a separate group of adolescent mice receiving the SSRI in their drinking water from PD35 to PD49. Figure 7 shows that on PD49 (i.e., after 15 days of FLX consumption), serum FLX was 2249.16 ± 185.91 ng/ml when administered at 250 mg/l in drinking water (n = 4).

## Discussion

The results of the present study highlight that exposure to the SSRI FLX, in juvenile female mice, leads to a long-term anxiety-like behavioral profile in adulthood, per the OFT, LDB, and EPM tests, without alterations in general locomotor activity (Figs. 2B, 4B). Specifically, when compared to the VEH-pretreated groups, adult female mice with FLX history, spent less time in the center of the OFT and the lighted area of the LDB, while spending more time inside the closed arms of the EPM (see Figs. 2A, 3A, 4A)—a behavioral response that, collectively, indicates that SSRI pretreatment mediates a long-term upward shift in baseline responses to anxiety-relevant situations^37^. Of note, these results agree with previous studies using similar behavioral tasks wherein early-life FLX induced an anxiety-like phenotype in adult male rodents^16–19^; critically, the current work expands these findings to female mice and further explores potential biochemical mechanisms of this effect.

Juvenile exposure to FLX, mediating an anxiety-like outcome in adulthood, is an interesting paradoxical finding since this SSRI is commonly prescribed for the management of anxiety disorders^38,39^. Indeed, under normal conditions, adult rodents chronically exposed to FLX display anxiolytic-like behavior^40^, or no long-term behavioral alterations at all^41^, mimicking human responses^42^. Such paradoxical behavioral profile, as a function of developmental SSRI exposure, indicates that adolescence is a vulnerable window for later-life adverse effects. Specifically, that chronic disruptions of serotonin functioning during this stage of development lead to enhanced reactivity to anxiety-related stimuli in adulthood^19^. As such, juvenile exposure to FLX may leave the organism in need of future pharmacological intervention to ameliorate these unwanted effects later in life, given that SSRIs are commonly prescribed for the management of anxiety. For this reason, we also evaluated whether FLX re-exposure in adulthood, in female mice with juvenile FLX history, would reverse the behavioral responses observed on the OFT, LDB, and EPM (see Fig. 1B). As expected, re-exposure to FLX in adulthood normalized responses across all behavioral tests (Figs. 2C, 3C, 4C). This behavioral profile, wherein FLX rescues early-life SSRI-induced alterations to anxiety-related situations, has been reported previously in male mice^18^ and rats^19^. This emphasizes that early-life antidepressant treatment renders both males and females in need of subsequent antidepressant re-exposure to normalize responses when encountering anxiety-inducing situations^37^, a critical advance considering the prevalence of mood disorders and SSRI prescription amongst females.

The molecular mechanisms by which juvenile FLX exposure mediates persistent anxiety-like responses in adulthood, and normalizes them upon re-exposure, are currently unknown. Acutely, and under normal conditions, FLX indirectly increases serotonin levels via the blockade of 5-HTTs. This global increase in serotonin tone, after repeated exposure, ultimately leads to serotonin receptor alterations^43,44^ and elevations in BDNF-related signaling molecules^45^. For this reason, we evaluated protein levels of 5-HTTs and BDNF’s TrkB receptor, as well as *key* down stream signaling molecules (ERK-CREB-BDNF) within the hippocampus and prefrontal cortex of adult female mice with juvenile FLX pretreatment. Interestingly, we found that adolescent FLX pre-exposure did not influence the expression of 5-HTTs or TrkB receptors in either brain region assessed (Figs. 5B, 6B). This was surprising given that previous studies in male animals have shown that developmental exposure to FLX mediates persistent alterations in the expression of 5-HTTs and TrkB, within these brain regions^18,22,46,47^. As such, the present null data on 5-HTT’s and TrkB receptors in female mice indicate that early-life SSRI exposure mediates divergent lasting changes in membrane bound receptors (5-HTT and TrkB) that are male^22^ but not female-specific (Figs. 5B, 6B); highlighting a long-term FLX-induced molecular signature between the sexes that results in anxiety-like behavior in both females and males. Subsequently, we also evaluated intracellular hippocampal and prefrontal cortex ERK-CREB-BDNF signaling markers, given that these downstream molecules also modulate affect-related behavior in intact adult rodents^25,30,48^. Here, we found that juvenile FLX pretreatment resulted in a long-term decrease in the phosphorylation of ERK2 and CREB within both brain regions (Fig. 5A, 6A, respectively), with additional decreases in total BDNF protein (both proBDNF and mBDNF) within the prefrontal cortex (Fig. 6A); molecular changes that likely underlie the female-specific anxiety-like behavioral phenotype observed in adulthood. Indeed, under normal conditions, acute decreases in BDNF^49^, ERK^50^, and CREB^51^ induce anxiety-like responses in preclinical models of anxiety^52,53^, with FLX rescuing these behavioral alterations via enhancement in the expression of these proteins. Importantly, this effect is observed in both animal models^30^, as well as in postmortem studies of patients who underwent SSRI treatment^54,55^, thus providing face and construct validity to our preclinical findings^56^.

Because adolescent SSRI exposure (PD35-49) resulted in the downregulation of hippocampal and prefrontal cortex ERK-CREB-BDNF signaling 21-days post treatment, we also evaluated how FLX re-exposure (PD70-84) would influence these signaling molecules in adulthood (PD85). In this case, we found that adult FLX re-exposure did not rescue the juvenile SSRI-induced hippocampal downregulation of pERK1/2 and pCREB at PD85 (Fig. 5C). However, within the prefrontal cortex, adult FLX re-exposure normalized pERK, pCREB, and t-proBDNF molecules, while significantly increasing total protein levels of mBDNF, when compared to controls (Fig. 6C). Taken together, these data pinpoint that the prefrontal cortex, but not the hippocampus, may underlie FLX’s ability to exert its anxiolytic-like effects in adult female mice with juvenile FLX history. Furthermore, these results emphasize that exposure to FLX mediates lasting changes in neuronal processes when administered during vulnerable periods of development, such as adolescence^57^, and that re-exposure to FLX in adulthood normalizes functioning via enhancement of prefrontal cortex plasticity processes. Supporting this notion, FLX reactivated neuronal plasticity and normalized functioning within the visual cortex of adult male mice that displayed visual circuitry imbalances, as a function of visual deficits during early periods of development. Likewise, improvements in the visual system were the result of FLX’s ability to increase the expression of BDNF within this system^58^, an effect we also find in adult females (Fig. 6C).

A limitation of this investigation is that we did not experimentally account for the role of the estrous cycle on the anxiety-like behavioral responses across our pharmacological conditions. Particularly because in naturally cycling adult female C57BL/6 mice, FLX’s anxiolytic-related effects on the EPM are dependent on the *estrus* phase of the cycle^59^. As such, future investigations are needed to dissect the relationship between the specific stages of the estrous cycle and adult responses to anxiety-related stimuli as a function of juvenile FLX pretreatment. Another caveat is that the mice utilized in this investigation are not categorized as an anxious strain, unlike BALB/c mice. Thus, experiments incorporating highly emotional strains, or undergoing stress-related models^60,61^ may exhibit different enduring FLX-induced mood-related traits. Lastly, the 250 mg/l dose of FLX in drinking water (for 15 days) resulted in serum levels (Fig. 7) that are significantly higher than the therapeutic dose range that is reported in humans (150–800 ng/ml)^62,63^. Yet, the plasma FLX concentrations in this investigation are comparable to those previously reported in adult male BALB/c mice receiving 25 mg/kg^40^; which also displayed lower avoidance-related behavior in the open field test^40^. Likewise, the FLX regimen implemented results in an antidepressant-like behavioral effect when screened in traditional preclinical models of despair in mice^14^. Specifically, both PD49 (adolescent) and PD84 (adult) female C57BL/6 mice display immobility decreases (traditionally interpreted as an antidepressant-like effect^64^) when evaluated on the tail suspension test after a 2-week exposure to 250 mg/l of FLX in their drinking water, without alterations in general locomotor activity (see Figs. 2B, 4B, as well as previous published work^14^). We therefore consider this a behaviorally relevant dose in rodents, and thus consider the effects of this dose on juvenile rodents to be applicable to the potential effects of FLX administered to adolescent patients in the clinic. Nevertheless, it will be critical to perform longitudinal studies in patients exposed to FLX during adolescence to determine whether there are subsequent effects on anxiety in these populations.

While FLX is regarded as a safe and efficacious pharmacological treatment for pediatric mood-related disorders^65^, the data from the present study uncovered enduring anxiety-related consequences, along with hippocampal and prefrontal cortex molecular alterations, as a function of its pre-exposure during adolescence in mice. Interestingly, our results further demonstrate that this persistent SSRI-induced anxiety-like response, in female C57BL/6 mice, can be rescued by re-exposure to FLX in adulthood—an effect that is likely explained by FLX’s ability to reverse the long-lived SSRI-induced molecular changes in the adult prefrontal cortex, but not the hippocampus. Although speculative, these findings may have important clinical implications, since they suggest that juvenile FLX exposure may render females reliant on SSRI treatment for sustained normalcy in later life—suggesting that once juvenile FLX treatment begins, potential need for lifetime management with SSRI medications could be a risk.

## Methods

### Animals

All procedures complied with the *Guide for the Care and Use of Laboratory Animals*^66^, the *Animal Research: Reporting *In Vivo* Experiments* (ARRIVE) guidelines^67^, and were approved by the Institutional Animal Care and Use Committee at The University of Texas at El Paso. Juvenile female C57BL/6 mice arrived at our laboratory at postnatal day (PD)-28 from Charles River Laboratory (Hollister, CA). Mice were housed in polypropylene cages (3–4 per cage) bedded with wood shavings, and with free access to food and water. The colony room was maintained at 21–23 °C on a 12-h light/dark cycle (lights on at 700 h). Mice were allowed to acclimate for 1-week before the start of the experiments.

### Drug treatment

Fluoxetine hydrochloride (FLX; Spectrum Chemicals, Gardena, CA, USA) was diluted (250 mg/l) in water (vehicle; VEH), and administered ad libitum in the drinking water (changed weekly) in light protected bottles (Model PC9RH8.5RD; Ancare, Bellmore, New York, USA). We chose this route of administration to reduce animal attrition due to tissue necrosis associated with chronic intraperitoneal injections^68^, and/or FLX-induced constipation^69^. The concentration of FLX in the water was selected based on previous work showing that this measure induces an antidepressant-like behavioral profile in rodent models of despair^14^, and it yields a dose close to 25 mg/kg^40^—considering that females and adolescents display increased metabolic rates when compared to males and adults, respectively^47,70,71^. To ascertain this, serum FLX levels were evaluated in a separate group of animals (Fig. 1C) via ultra performance liquid chromatography (see Supplemental Information for details)^72,73^.

### Experimental design

To evaluate whether juvenile FLX exposure mediates an enduring anxiety-like outcome, along with altered molecular changes in 5-HTT/BDNF signaling, adolescent female C57BL/6 mice were randomly assigned to receive VEH or FLX for 15 consecutive days (PD35–49; Fig. 1A). This regimen was chosen because it resembles the timeframe of development for human adolescence^74^. After a 21-day resting period (PD70), separate cohorts of animals were either euthanized (for tissue collection) or tested in behavioral tasks designed to assess anxiety-like behavior^37,75^—the open field test (OFT), light/dark box (LDB), and elevated plus maze (EPM). Because adolescent FLX exposure increased reactivity to anxiety-inducing situations in adulthood across all behavioral paradigms (Figs. 2A, 3A, 4A), we designed a follow-up experiment (Fig. 1B) to evaluate whether FLX re-exposure in adulthood would normalize the enduring FLX-induced molecular (Figs. 5A, 6A) and behavioral alterations observed. Specifically, juvenile mice received FLX or VEH for 15 days (PD35-49), matching FLX pretreatment during adolescence as in our initial experiment. However, in this case, after the 21-day break, we re-exposed the animals to FLX for two weeks in adulthood (PD70-84). Twenty-four hours after FLX re-exposure (PD85), separate groups of animals were euthanized (for tissue collection) or tested on the OFT, LDB, and EPM (see Supplementary Table 2 for experimental groups). A video tracking program (EthovisionXT; Noldus, Leesburg, VA) was used to record behavior across all tasks.

### Open field test (OFT)

The OFT is commonly used to evaluate general locomotor activity, as well as exploratory and anxiety-like behavior^37^. In this test, under red lighting conditions (~ 30 lx), rodents were placed inside a white square box (40 cm in length × 40 cm in width × 40 cm in height) for a total of 5 min^19^. Initially, rodents mostly explore the periphery of the box, but eventually start exploring the center area (18 cm × 18 cm). Total distance traveled (cm) and time (sec) spent in the center of the box were recorded. Less time spent in the center of the arena was interpreted as an anxiety-like phenotype^76^.

### Light/dark box (LDB)

The LDB apparatus is comprised of two interconnected chambers (each 20 cm in width × 40 cm in length × 35 cm in height). One chamber is black and enclosed, while the other one is white and not enclosed (Model 63,101; Stoelting, Wood Dale, IL). This behavioral paradigm takes advantage of rodents’ innate instinct to avoid brightly illuminated areas, as well as to explore novel environments, thus creating an internal conflict described as anxiety-like behavior^77,78^. Under red lighting conditions (~ 30 lx), at the start of the experiment, animals were placed in the dark compartment and allowed to move freely between the two chambers for 5 min. The latency (sec) to enter the light side of the apparatus, as well as the total time spent in it, were the dependent variables. Less time spent in the light side of the box was interpreted as an anxiety-like behavioral response.

### Elevated plus maze (EPM)

This is a classic task used to evaluate anxiety-like behavior in rodents^25,37,79^. Briefly, the animals were tested in a plus-shaped maze (under red light conditions; ~ 30 lx) that was elevated 50 cm from the floor. The apparatus has two open arms (35 cm in length × 5 cm in width) and two closed arms (35 cm in length × 5 cm in width × 15 cm in height; Model 60140; Stoelting, Wood Dale, IL). Animals were placed in the center of the maze facing an open arm and allowed to freely explore the apparatus for 5 min. Time (sec) spent in the closed arms, as well as total distance travelled (cm) were recorded^15^. More time spent in the closed arms was interpreted as an anxiety-relevant phenotype^80^.

### Western blotting

Twenty-one days after the last day of FLX exposure (for adolescent pre-exposure group; Fig. 1A) or 24 h after FLX re-exposure (for the adolescent pretreated and adult re-exposure group; Fig. 1B), brains were rapidly extracted, and hippocampus and prefrontal cortex were microdissected on dry ice^81^ and stored at − 80 °C until assayed. Tissue was homogenized by sonication in 300 μL of tissue protein extraction buffer (ThermoFisher Scientific, #78510) supplemented with protease and phosphatase inhibitors (Roche, #04693116001, #04906845001), and cleared by centrifugation at 14,500 rpm for 10 min. Supernatants were collected as total homogenate, and protein concentration was determined via the bicinchoninic acid method (ThermoFisher Scientific, #23225), using bovine serum albumin as standard. Samples were dissolved in Laemmli buffer (BioRad, #1610737) containing 5% β-mercaptoethanol (BioRad, #1,610,710), and equal amounts of hippocampal and cortical homogenates were subjected to electrophoresis using 8–16% gradient pre-cast gels (BioRad, #5678105), followed by electrotransfer onto polyvinylidene difluoride membranes (BioRad, #1704159). The membranes were blocked in 5% non-fat dry milk dissolved in Tris buffered saline with Tween (TBST: 100 mM Tris–HCl, pH 7.4, 150 mM NaCl, and 0.05% Tween 20), and incubated overnight at 4 °C with primary antibodies (1:1000) dissolved in blocking buffer (see Supplementary Table 3 for list of antibodies). Membranes were washed with TBST and incubated with appropriate horseradish peroxide-conjugated secondary antibodies (Cell Signaling Technology, anti-rabbit 7074S, anti-mouse, #7076S; Abcam, anti-goat #ab6741). Protein bands were visualized with the enhanced chemiluminescence technique, using the Clarity Western Substrate kit (BioRad, #1705061). Membranes were subsequently stripped with Restore Stripping Buffer (ThermoFisher Scientific, #46430) and re-probed with anti-tubulin antibody to serve as loading control. Protein band densitometry was analyzed with ImageJ software (National Institutes of Health).

### Statistical analysis

Separate groups of female mice were randomly assigned to receive VEH or FLX during adolescence (long-term condition; groups 1, 2, 3, and 7 on Supplementary Table 2). Additional groups of female mice were assigned to receive VEH or FLX during adolescence and again in adulthood (re-exposure condition; groups 4, 5, 6, and 8 on Supplementary Table 2). Data were analyzed using two-tail Student's t tests. Data are presented as mean ± SEM. Statistical significance was defined as *p* < 0.05.

## Supplementary Information

Supplementary Information
