# Supplementary material for: Adolescent fluoxetine treatment mediates a persistent anxiety-like outcome in female C57BL/6 mice that is ameliorated by fluoxetine re-exposure in adulthood
Source: Sci Rep. 2021 Apr 8;11:7758. doi: 10.1038/s41598-021-87378-6 (PMC8032660; doi:10.1038/s41598-021-87378-6)
Supplement: Supplementary file 1 — Supplementary Information [file 41598_2021_87378_MOESM1_ESM.docx]

SUPPLEMENTAL MATERIAL

**Adolescent fluoxetine treatment mediates a persistent anxiety-like outcome in female C57BL/6 mice that is ameliorated by fluoxetine re-exposure in adulthood**

^1^Francisco J. Flores-Ramirez, ^1^Anapaula Themann, ^1^Jorge A. Sierra-Fonseca, ^1^Israel Garcia-Carachure, ^1^Samuel A. Castillo, ^1^Minerva Rodriguez, ^1^Omar Lira, ^1^Joshua Preciado-Piña, ^2^Brandon L. Warren, ^3^Alfred J. Robison, and ^1^*Sergio D. Iñiguez

^1^Department of Psychology, The University of Texas at El Paso, El Paso, TX; ^2^Department of Pharmacodynamics, University of Florida, FL; ^3^Department of Physiology, Michigan State University, East Lansing, MI

*Corresponding Author: Sergio D. Iñiguez, Ph.D. (sdiniguez@utep.edu), Department of Psychology, The University of Texas at El Paso, 500 West University Avenue, El Paso, TX, 79968. Phone: 915-747-5769, Fax: 915-747-6553.

**Analysis of fluoxetine (FLX) in blood serum samples**

Blood was collected in blood collection tubes (Vacuette tube 2 ml K2E K2EDTA, #454428, Greiner Bio-One) when mice were euthanized after FLX exposure (PD49; see Fig. 1C). The serum fraction was prepared by centrifugation at 3000 RPM for 10 min. After centrifugation, the supernatant was transferred to a new tube and immediately stored at -80 °C until assayed. A bioanalytical method reported by Kertys et al.,^71^ was partially modified for the quantification of FLX in mouse serum samples using Waters Xevo TQ-S Micro triple quadrupole mass spectrometer (Milford, MA, USA). Chromatographic separation was achieved on Acquity I-Class ultra performance liquid chromatography using a mobile phase that consisted of water and acetonitrile containing 0.1% formic acid and Waters Acquity BEH C18 column (1.7 μm, 2.1 × 50 mm). Mobile phase compositions and flow rates were implemented directly from the method reported by Kertys and colleagues^71^. Verapamil was used as an internal standard. Ionization of FLX and internal standard was achieved using electrospray ionization in positive mode. A multiple reaction monitoring (MRM) mode was implemented for the detection, and compound parameters are mentioned in Supplementary Table 1 (below). Serum samples (20 µl) were quenched using acetonitrile (60 µl) containing internal standards (10 ng/ml) for the removal of endogenous compounds and extraction of fluoxetine. Test samples collected from FLX dosed mice were analyzed along with freshly prepared eight-point calibration standards (10, 20, 50, 100, 200, 300, 400, and 500 ng/ml) and quality control samples (10, 30, 250, and 450 ng/ml). Dilution integrity (20X) was performed to analyze test samples after dilution with drug-free mouse serum. The method was linear for a concentration range of 10-500 ng/ml, and the accuracy and precision of the method were within the Food and Drug Administration specified limits^72^.

Supplementary Table 1. Compound parameters for fluoxetine and internal standards

| Compound | Mass transition (*m/z*) | Cone Voltage (V) | Collision Energy (V) |
| --- | --- | --- | --- |
| Fluoxetine (quantifier) | 309.97 > 148.16 | 6 | 6 |
| Fluoxetine (qualitifier) | 309.97 > 91.14 | 6 | 80 |
| Verapamil (internal standard) | 455.27 > 150.10 | 4 | 42 |

| Supplementary Table 2. Experimental Groups | | | | | | | |
| --- | --- | --- | --- | --- | --- | --- | --- |
| Group | Treatment | n | Age | Interval | Procedure I | Procedure II | Data |
| 1 | VEH | 10 | PD35-49 | 21-days | OFT  (PD70) | – | Fig. 2A-B |
|  | FLX | 10 |  |  |  |  |  |
| 2 | VEH | 10 | PD35-49 | 21-days | LDB  (PD70) | – | Fig. 3A-B |
|  | FLX | 10 |  |  |  |  |  |
| 3 | VEH | 10 | PD35-49 | 21-days | EPM  (PD70) | – | Fig. 4A-B |
|  | FLX | 10 |  |  |  |  |  |
| 4 | VEH | 10 | PD35-49 | 21-days | VEH or FLX Re-exposure (PD70-84) | OFT  (PD85) | Fig. 2C-D |
|  | FLX | 10 |  |  |  |  |  |
| 5 | VEH | 10 | PD35-49 | 21-days | VEH or FLX Re-exposure (PD70-84) | LDB  (PD85) | Fig. 3C-D |
|  | FLX | 10 |  |  |  |  |  |
| 6 | VEH | 10 | PD35-49 | 21-days | VEH or FLX Re-exposure (PD70-84) | EPM  (PD85) | Fig. 4C-D |
|  | FLX | 10 |  |  |  |  |  |
| 7 | VEH | 12 | PD35-49 | 21-days | Western blot Long-Term (PD70) | – | Fig. 5A-B Fig. 6A-B |
|  |  |  |  |  |  |  |  |
|  | FLX | 12 |  |  |  |  |  |
| 8 | VEH | 12 | PD35-49 | 21-days | VEH or FLX (PD70-84) | Western blot | Fig. 5C-D Fig. 6C-D |
|  |  |  |  |  |  | Re-exposure |  |
|  | FLX | 12 |  |  |  | (PD85) |  |
| 9 | FLX | 4 | PD35-49 | – | Blood Collection | – | Fig. 7 |
|  |  |  |  |  |  |  |  |

EPM, elevated plus maze; FLX, fluoxetine; LDB, light-dark box; OFT, open field test; PD, postnatal day; VEH, vehicle (water).

| Supplementary Table 3. Western Blot Primary Antibodies | | | |
| --- | --- | --- | --- |
| Antibody | Source | Company | Product # |
| 5-HTT | Goat | Abcam (Burlingame, CA) | ab130130 |
| BDNF | Rabbit | Abcam (Burlingame, CA) | ab108319 |
| p-ERK 1/2 | Rabbit | Cell Signaling (Danvers, MA) | 4370 |
| t-ERK 1/2 | Mouse | Cell Signaling (Danvers, MA) | 4696 |
| p-CREB | Rabbit | Cell Signaling (Danvers, MA) | 9198 |
| t-CREB | Rabbit | Cell Signaling (Danvers, MA) | 9197 |
| Trk-B | Rabbit | Cell Signaling (Danvers, MA) | 4603 |
| α-Tubulin | Mouse | Sigma-Aldrich (St. Louis, MO) | T6199 |

Supplementary Figure S1.


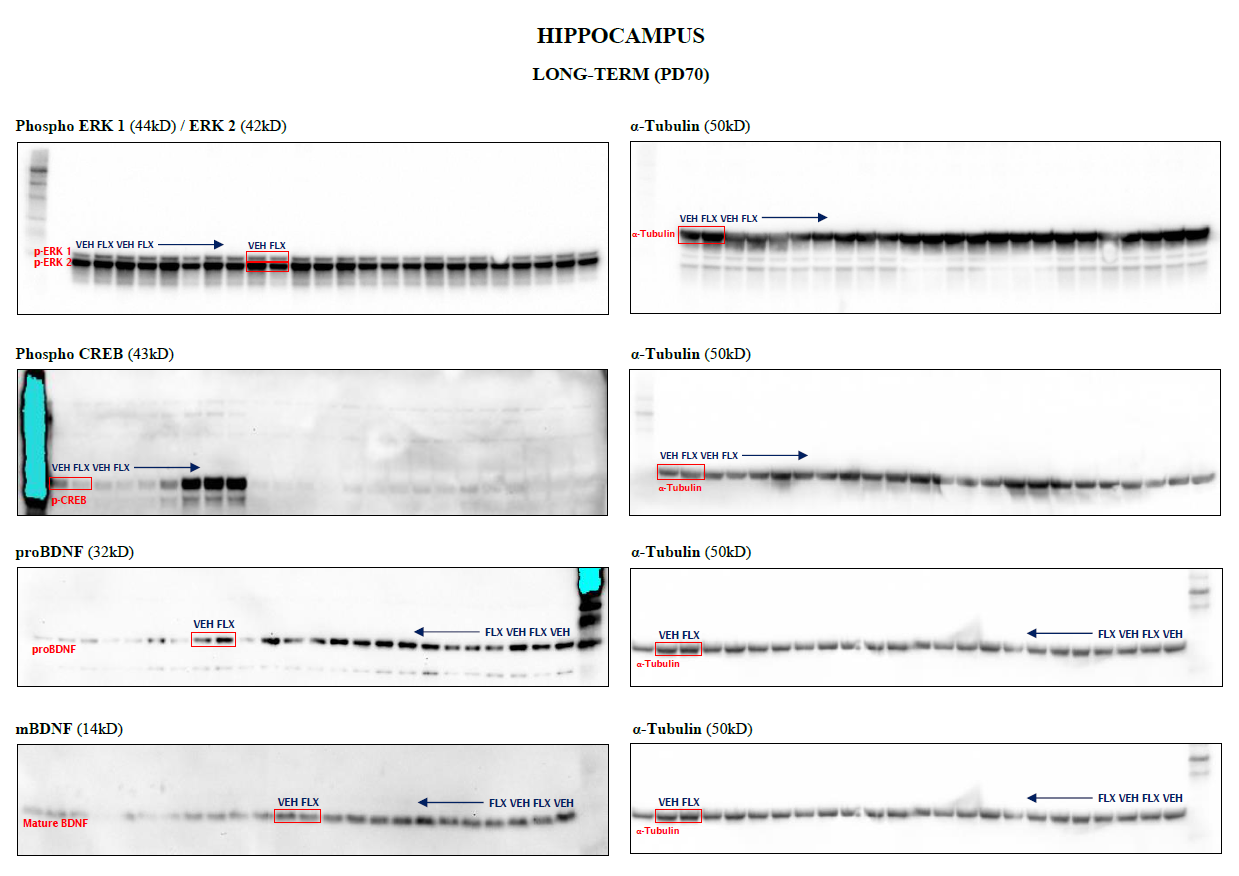


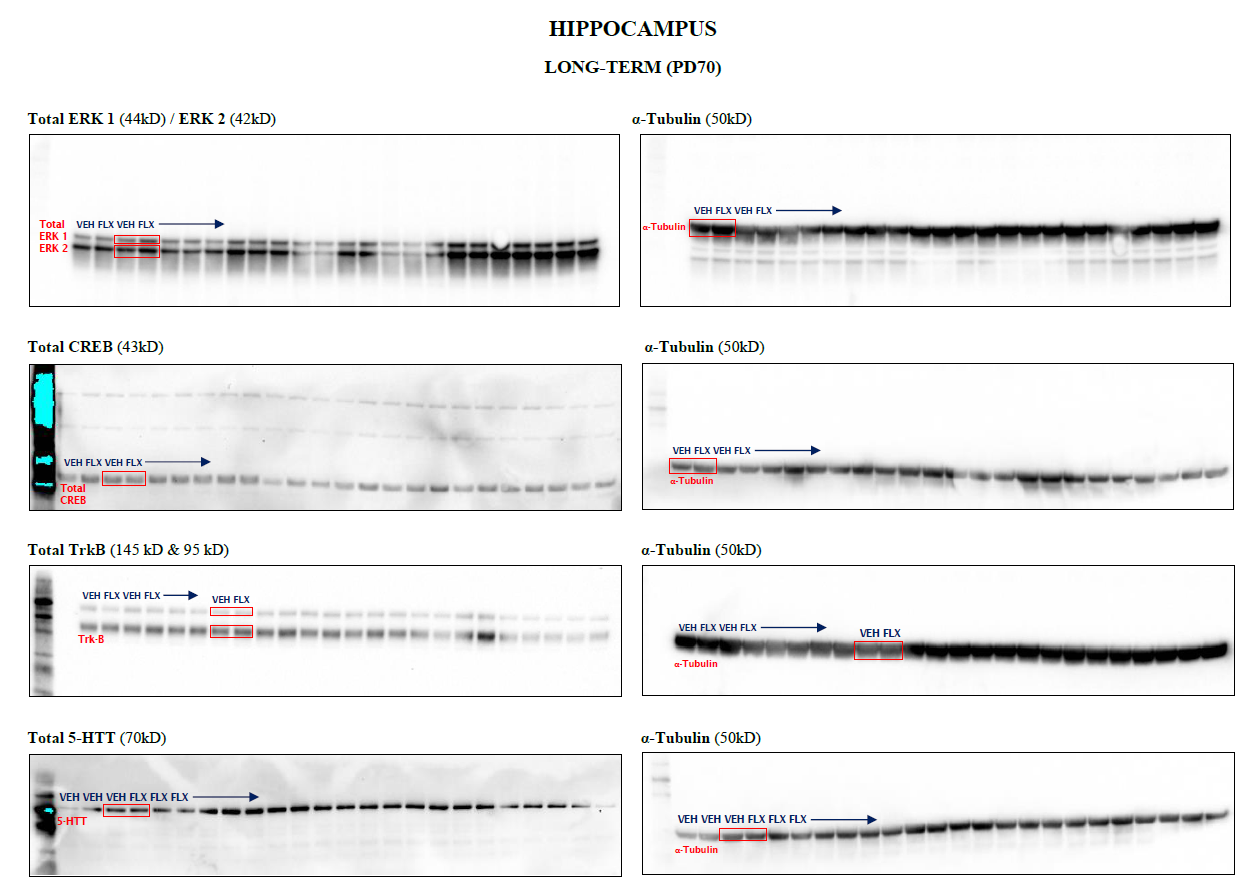


**
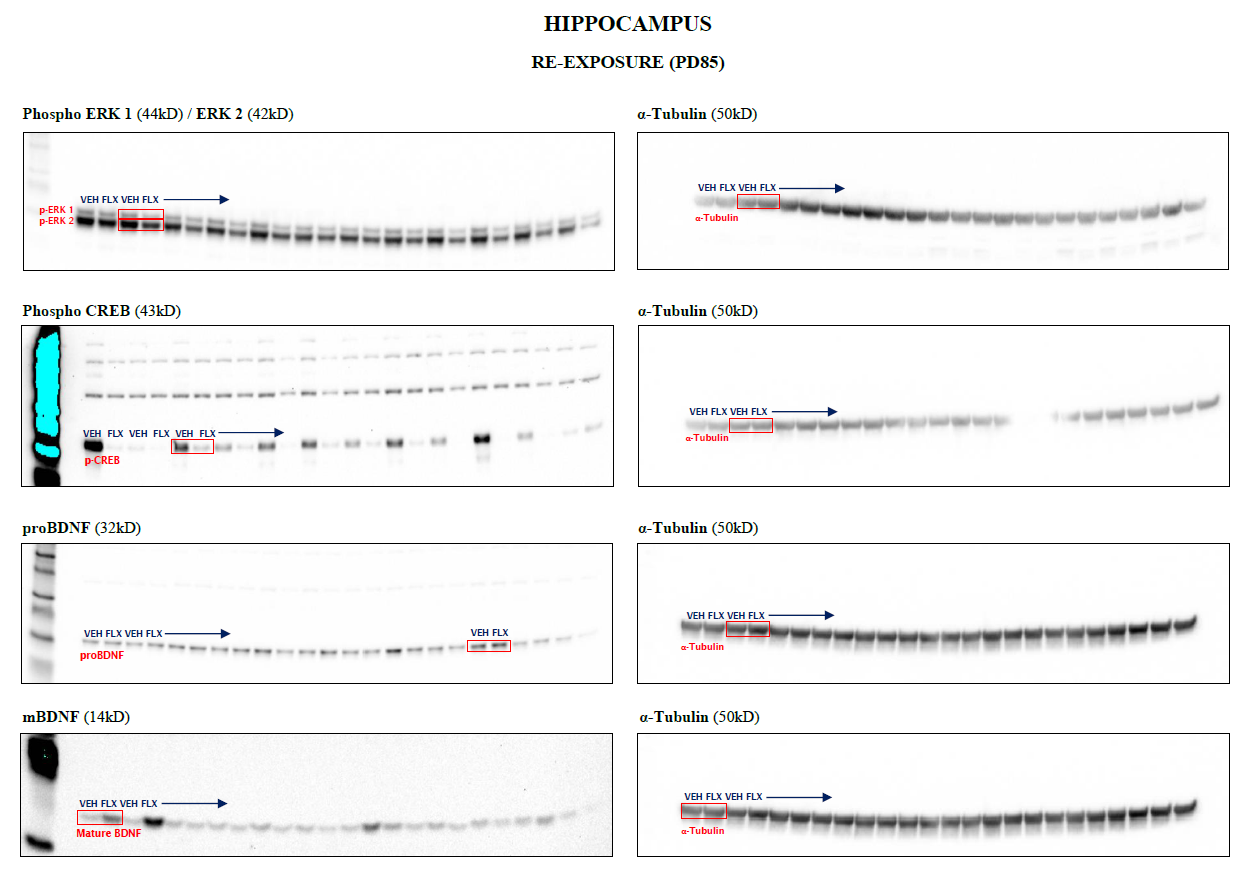
**

**
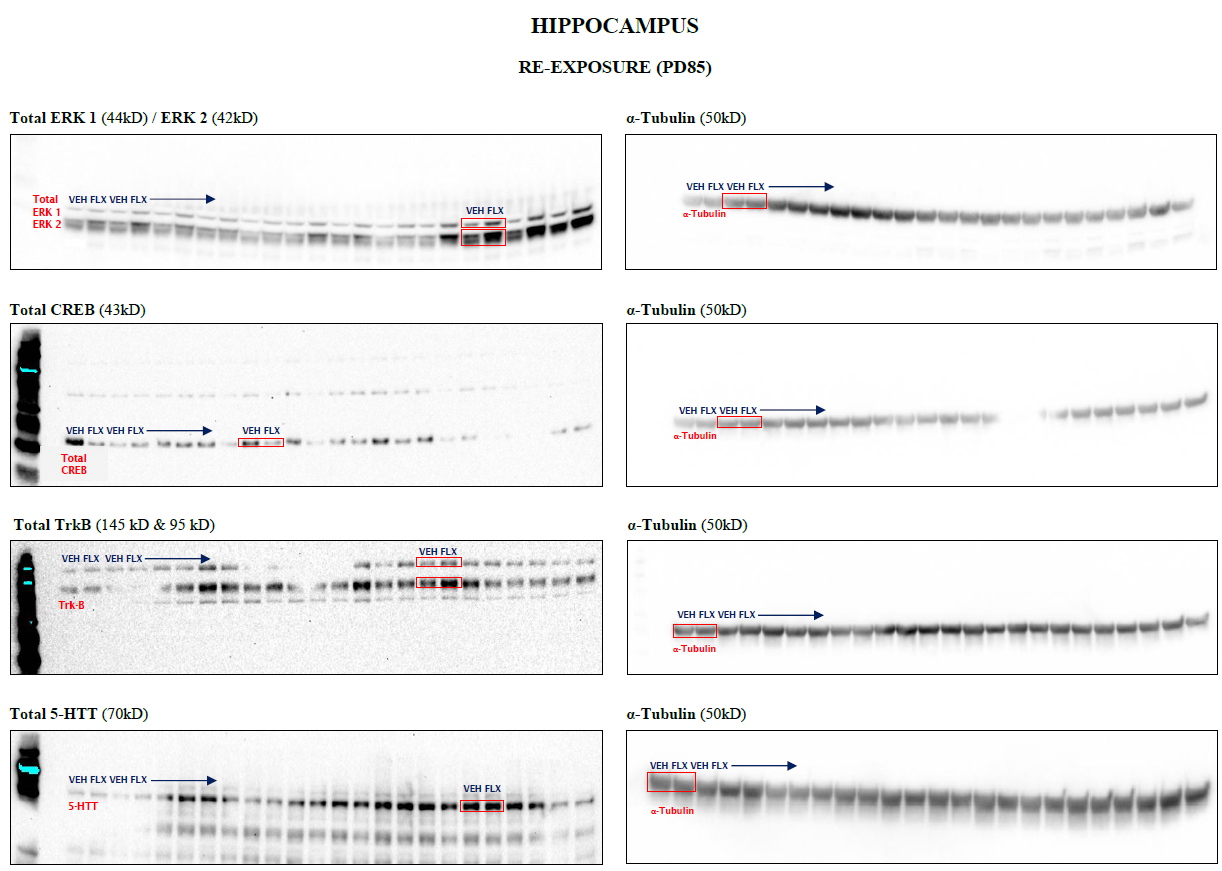
**

**Figure S1.** Full blots of ERK1/2, CREB, TrkB, BDNF, and 5-HTT hippocampal proteins. Arrow indicates loading direction. Blots were imaged using the ChemiDoc XRS+ imaging system (Bio-Rad, Hercules, CA).

Supplementary Figure S2.


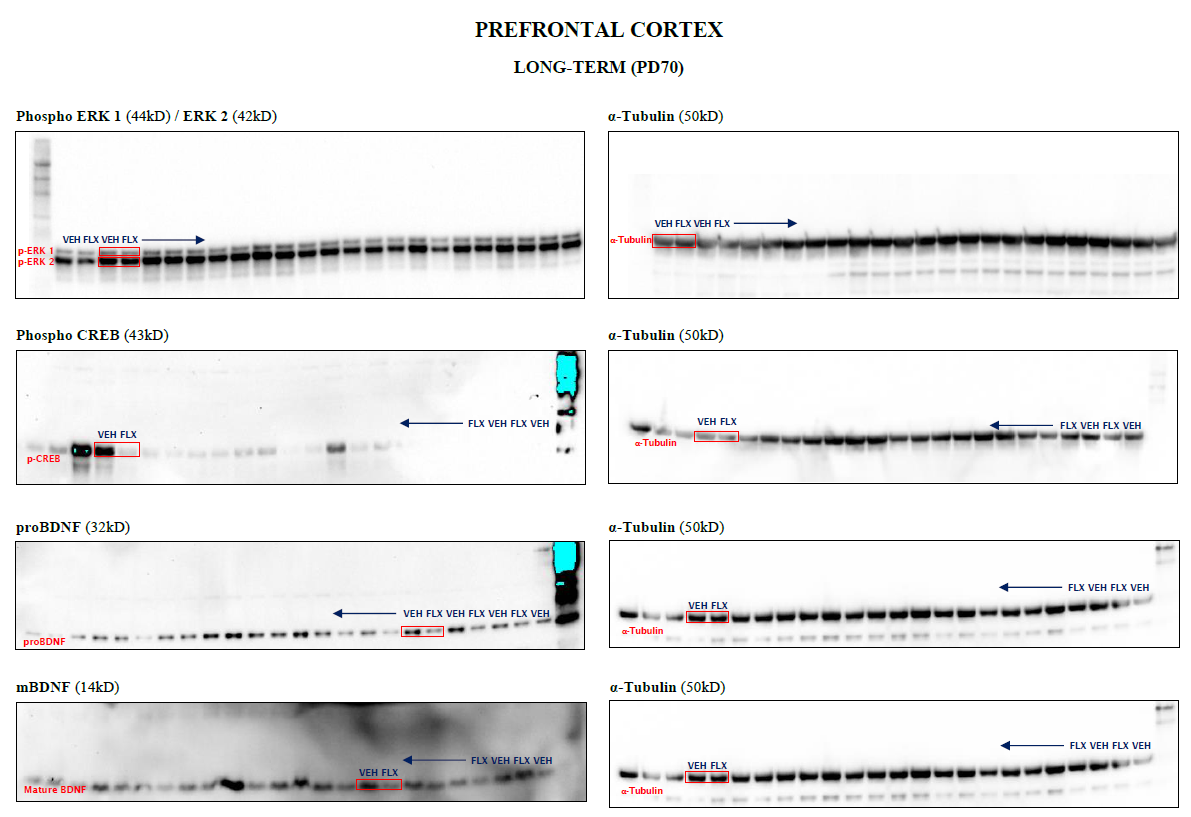


**
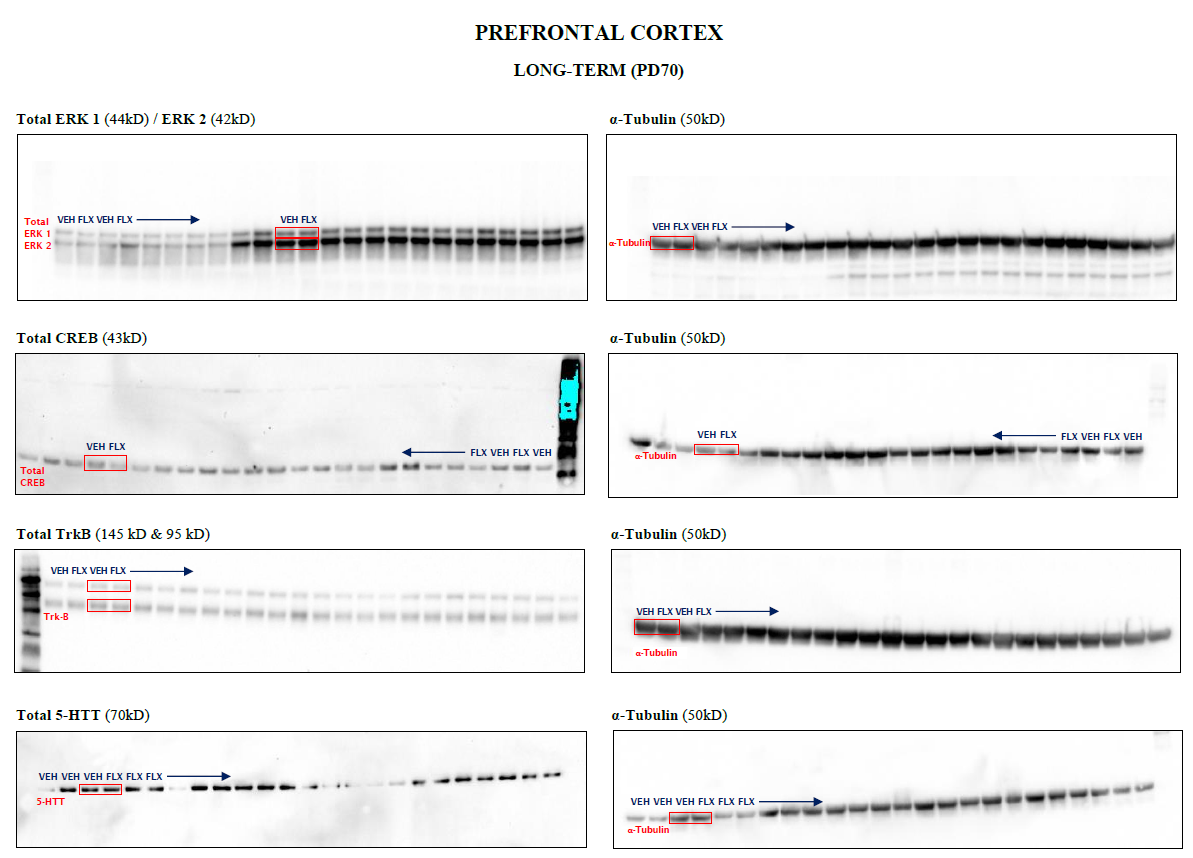
**

**
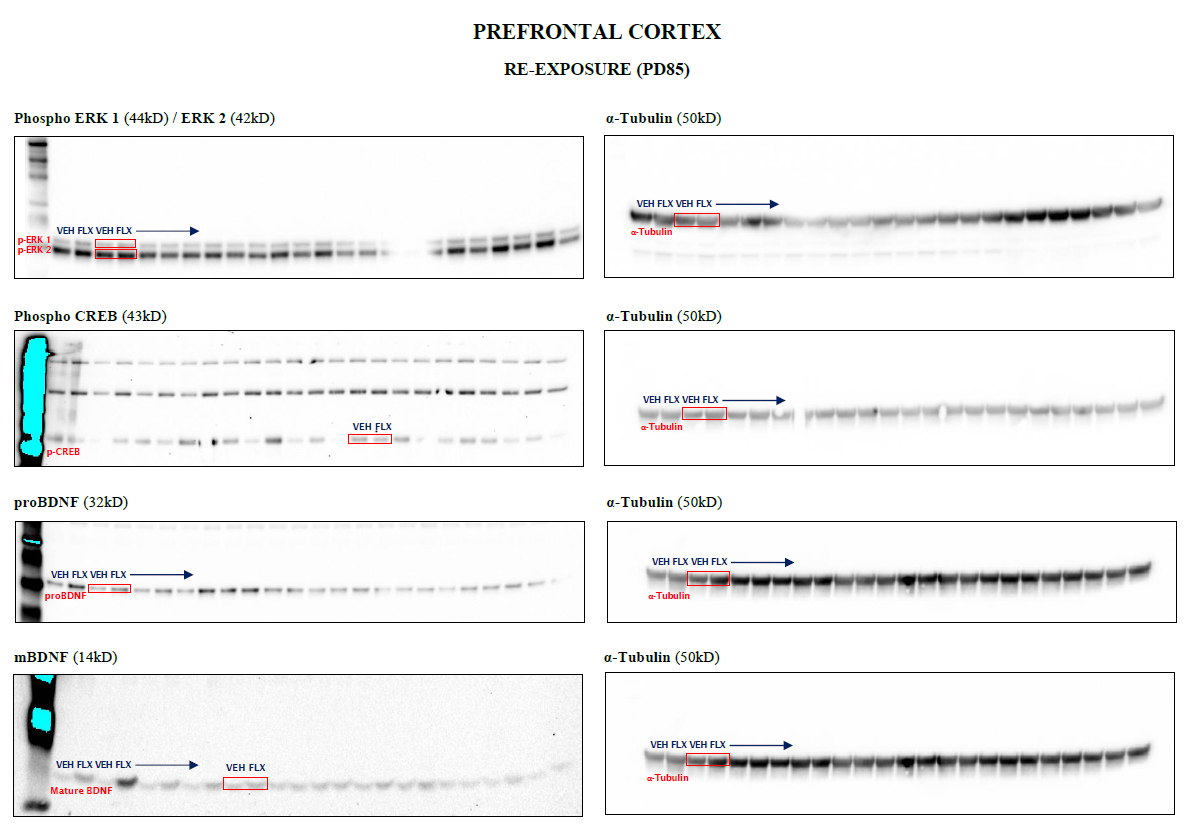
**

**
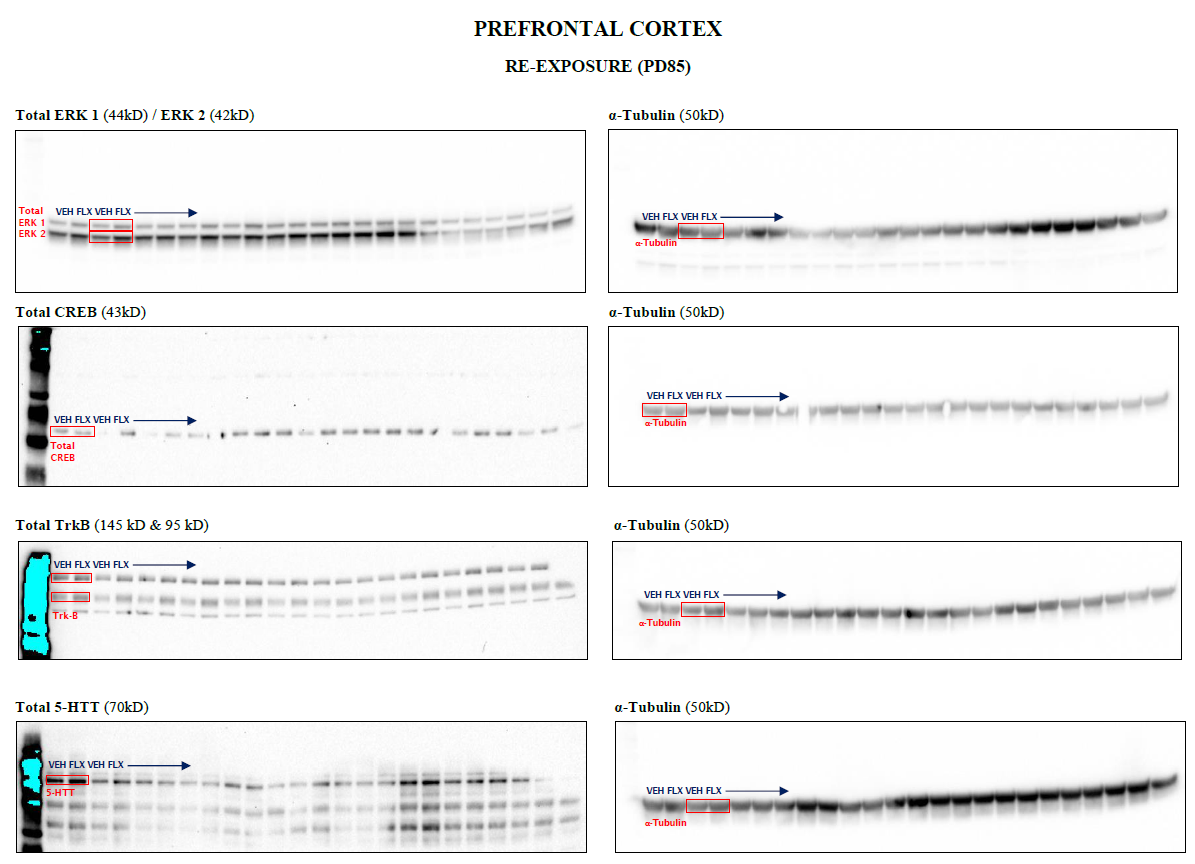
**

**Figure S2.** Full blots of ERK1/2, CREB, TrkB, BDNF, and 5-HTT prefrontal cortex proteins. Arrow indicates loading direction. Blots were imaged using the ChemiDoc XRS+ Imaging System (Bio-Rad, Hercules, CA).
